# Bioactivity and Thermal Stability of Collagen–Chitosan Containing Lemongrass Essential Oil for Potential Medical Applications

**DOI:** 10.3390/polym14183884

**Published:** 2022-09-17

**Authors:** Maria Râpă, Traian Zaharescu, Laura Mihaela Stefan, Carmen Gaidău, Ioana Stănculescu, Rodica Roxana Constantinescu, Maria Stanca

**Affiliations:** 1 Faculty of Materials Science and Engineering, University Politehnica of Bucharest, 313 Splaiul Independentei, 060042 Bucharest, Romania; 2 INCDIE ICPE CA, 313 Splaiul Unirii, P.O. Box 149, 030138 Bucharest, Romania; 3 National Institute of R&D for Biological Sciences, 296 Splaiul Independentei, 060031 Bucharest, Romania; 4 The National Research & Development Institute for Textiles and Leather-Division Leather and Footwear Resesarch Institute (ICPI), 93 Ion Minulescu Street, 031215 Bucharest, Romania; 5 Horia Hulubei National Institute of Research and Development for Physics and Nuclear Engineering, 30 Reactorului Street, 077125 Magurele, Romania; 6 Department of Physical Chemistry, University of Bucharest, 4–12 Regina Elisabeta Boulevard, 030018 Bucharest, Romania

**Keywords:** collagen hydrolysate, chitosan, lemongrass essential oil, gamma irradiation, chemiluminescence, radical scavenging activity, in vitro cytotoxicity, antimicrobial property

## Abstract

Bioactive collagen–chitosan–lemongrass (COL–CS–LG) membranes were prepared by casting method and analyzed for potential biomedical applications. For COL–CS–LG membranes, LG essential oil release, antioxidant properties, in vitro cytotoxicity and antimicrobial assessments were conducted, as well as free radical determination after gamma irradiation by chemiluminescence, and structural characteristics analysis through Attenuated Total Reflection–Fourier Transform Infrared Spectroscopy (ATR–FTIR) and Differential Scanning Calorimetry (DSC). The evaluation of non-isothermal chemiluminescence after gamma radiation exposure to COL–CS–LG membranes revealed a slowing down of the oxidation process at temperatures exceeding 200 °C, in correlation with antioxidant activity. Antimicrobial properties and minimum inhibitory concentrations were found to be in correlation with cytotoxicity limits, offering the optimum composition for designing new biomaterials.

## 1. Introduction

The volume of requests for medical devices fabricated from natural polymers, such as animal-derived proteins that mimic the extracellular matrix (ECM) of tissues and organs, and marine animal polysaccharides, together with the use of alternative antimicrobial agents over conventional antibiotics, is increasing constantly. Moreover, achieving good physical–chemical features, antimicrobial and biocompatibility characteristics, as well as high degradation stabilities of materials designed for healthcare purposes subjected to radio-sterilization, are major concerns among researchers [1,2].

Collagen (COL) is comprised of amino acids, glycine, proline and hydroxyproline, which together form a triple-helix structure able to support the extracellular space of connective tissues. It is an essential constituent of the dermis, facilitating the acceleration of wound closure assisted by minimum scar production [3,4]. The main drawbacks of collagen are thermal degradation during processing and destroying of the triple-helix during extraction [5]. To overcome these main deficiencies of collagen, its combination with chitosan [6,7], and their mixture with organic and inorganic additives or bioactive compounds [5,8,9,10], has been recommended for tissue engineering applications [11,12]. Collagen hydrolysate loaded with thyme essential oil (*Thymus vulgaris*) and oregano essential oil (*Origanum vulgare*), respectively, were prepared by Berechet et al. [13] as a sustainable alternative for more expensive native collagen loaded with synthetic antimicrobials with pathogen resistance and inflammatory potential.

Chitosan (CS) (*N*-acetyl 1,4, β-d-glucopyranosamine) is a natural biopolymer produced from chitin [14], and is known as the second most plentiful polysaccharide after cellulose, showing regenerative and tissue reconstruction properties [15,16]. Due to favorable properties such as biodegradability, biocompatibility, film-forming, non-toxicity and antimicrobial activity, chitosan is exploited as a promising material for numerous biomedical applications.

Examples of the use of chitosan as a biomaterial are related to the fabrication of membranes as a transdermal drug release system [17,18], wound dressings [19,20] and tissue engineering [15,21,22]. Chitosan has been mixed with postbiotics of *L. reuteri* with the purpose of accelerating cutaneous wound healing [23]; forming therapeutic agents, such as nitric oxide (NO), for the treatment of MRSA-biofilm-infected wounds [24]; and oxidizing carboxymethyl cellulose (OCMC) [25], arginine and gold nanoparticles (NPs) to speed up wound healing [26]. Membranes, casted films and nanofibers produced by electrospinning can be efficiently fabricated from CS and its derivatives [27]. Collagen–chitosan-based membranes with improved mechanical properties, optical transmittance, hydrophilicity and attractive properties for cellular adhesion and proliferation have been successfully prepared for tissue engineering applications [6]. The increase in the bioactivity of collagen–chitosan mixtures has been investigated by Râpă et al. [9], who incorporated lemon balm (*Melissa officinalis* L.) and dill (*Anethum graveolens* L.) essential oils into solutions. The authors highlighted the antimicrobial activity against important bacterial strains, as well as the in vivo biocompatibility, of the nanofibers based on collagen hydrolysate mixed with aqueous chitosan.

Plant extracts, particularly essential oils, constitute a promising replacement for synthetic drugs, attributable to their antibacterial, antifungal, anti-inflammatory, and antioxidant features [28]. Among them, lemongrass (LG) essential oil (*Cymbopogon flexuosus*) has been most studied due to its demonstrated antimicrobial and insecticidal effects, accredited to the main citral (neral and geranial isomers) and geraniol bioactive compounds that make up 71.8–90% of its composition [29,30,31,32]. LG is used in the treatment of infections caused by polymicrobial biofilms [33] and in veterinary medicine [34]. Pan et al. [35] reported the antioxidant and anticancer activities of lemongrass conferred by the chemical structure of flavonoid compounds. Innovative patches or gauzes for wound care were prepared by encapsulation of 1 or 5% *v/v* of lemongrass essential oil into cellulose acetate (CA) fibrous mats by Kiakos et al. [36]. Additionally, myrcene and limonene are other aromatic bioactive compounds found in LG essential oil, which recommend its use as a functional ingredient for food and drink applications [30,37,38,39]. Despite the favorable properties of LG essential oil, its main drawbacks refer to its high volatility, low water solubility and poor resistance to oxidation [40]. Furthermore, studies related to dermal fibroblast cells [31] and the antiproliferative activity of cancer cells [41] exposed to LG essential oil showed that cytotoxicity calculated as relative cell viability (%) is dependent on the concentration of LG essential oil.

The aim of this study is to establish the optimal amount of LG essential oil that can be incorporated into collagen–chitosan natural polymers by the casting method, without diminishing the in vitro biocompatibility and antimicrobial activity, while ensuring high antioxidant and release properties. Therefore, the membrane containing 0.7% LG essential oil was further investigated for its resistance to gamma radiation in the sterilization of wound patches. The effect of irradiation on the structural and thermal properties of COL–CS–LG 0.7% was also evaluated by ATR–FTIR and DSC analyses. The presence of 0.7% LG essential oil in the collagen–chitosan membrane led to the formation of eco-friendly transparent material with enhanced performance associated with biocompatibility and antimicrobial activity, as well as thermal resistance to oxidation, and the suppression of radicals generated by gamma radiation, which recommends its use for potential medical applications. Original results relating to the chemiluminescence spectroscopy analysis are reported, highlighting the oxidative processes that developed after the exposure of the COL–CS–LG 0.7% membrane to gamma radiation.

## 2. Materials and Methods

### 2.1. Materials

Collagen hydrolysate (COL) was prepared from bovine skin at the Leather Research Department of ICPI (Bucharest, Romania) by chemical–enzymatic hydrolysis [42,43,44] and were freeze dried (DELTA 2-24 LSC, Osterode am Harz, Germany).

Chitosan (CS) [(C_6_H_11_O_4_N)_n_], highly viscose in the form of crystals, is characterized by a viscosity of 1267 MPa·s and a sulphated ash content of 0.2% (Sigma-Aldrich, Darmstadt, Germany).

Lemongrass (LG) essential oil (*Cymbopogon flexuosus*) is rich in geraniol and citronellol, which are antiseptics, anti-inflammatories, venous and cutaneous tonics, vasodilators and blood circulation stimulators. LG essential oil was purchased from Solaris Plant (Bucharest, Romania). Dimethyl sulfoxide (DMSO) of analytical grade (Scharlau, Scharlab S.L., Sentmenat, Spain) was used as a dispersing solvent for LG essential oil.

The following reagents used in the tests were of analytical quality and/or suitable for use in microbiology: water; tryptic soy broth culture medium (TSB); tryptone soy agar culture medium (TSA); enumeration agar (EA); culture medium of nutrient broth (NB); soybean casein digest lecithin polysorbate 80 medium (SCDLP); sabouraud dextrose agar medium (SDA); microorganism strains of *Staphylococcus aureus* ATCC 6538, *Escherichia coli* ATCC 10536, *Candida albicans* ATCC 10231 (Mediclim, Otopeni, Romania).

### 2.2. Sample Preparation

A solution of COL–CS, with a ratio between dry components of 2:1, was prepared by dissolving COL in distilled water (at a concentration of 57% (*w*/*v*)). Then, this solution was mixed with a 1.5% (*w*/*v*) solution of CS prepared in 1% (*v*/*v*) acetic acid under magnetic stirring at 800 rpm, and at 90 °C for 3 h. Subsequently, 5.3 mL of LG essential oil were dissolved in 1 mL DMSO under vigorous stirring at 800 rpm for 30 min. To obtain the collagen–chitosan–lemongrass membranes, different amounts of LG essential oil solution were added to the fixed 30 mL collagen–chitosan solution; the concentrations of LG essential oil (*v*/*v*) were 0%, 0.1%, 0.3%, 0.7% and 1%. The membranes, designated as COL–CS–LG 0.1%, COL–CS–LG 0.3%, COL–CS–LG 0.7% and COL–CS–LG 1%, were obtained through a casting method of ternary mixtures in glass Petri dishes. After drying the membranes (48 h at laboratory temperature), they are stored in the Petri dishes, covered with the cap, and kept in desiccator before characterization analysis. The COL–CS membrane without LG essential oil was also prepared as a blank sample.

### 2.3. Investigation Methods

#### 2.3.1. Physicochemical Characterization of Collagen and Qualitative Assessment of Membranes

The main characteristics of the collagen hydrolysate powder were determined for volatile matter (SR EN ISO 4684:2006), total ash content (SR EN ISO 4047:2002), total nitrogen and protein content (SR EN ISO 5397:1996), aminic nitrogen and molecular weight (Sorensen method), electrical conductivity (SR EN 2788:1997) and pH of 10% solution (STAS 8619/3:1990).

Transmittance of the obtained COL–CS–LG membranes was assessed using the Orion™ AquaMate 8000 UV/VIS spectrophotometer (Thermo Fisher Scientific, Madison, WI, USA) in the range of wavelengths from 200 to 800 nm.

The cumulative release of LG essential oil from the COL–CS membranes was also investigated through UV/VIS spectrometry. A predetermined LG essential-oil-specific calibration curve (0–1 mg mL^−1^) in DMSO was performed (y = 3.018x + 1.05, R^2^ = 0.9389). The weighed COL–CS–LG membranes were immersed in 10 mL DMSO and mixed at room temperature at predetermined time intervals (5 min, 20 min and 60 min). The absorbance of supernatant was monitored at 258 nm, which is the highest absorption peak of LG essential oil, using a 1-cm (path width) quartz cell, and the cumulative release of LG essential oil was estimated according to Equation (1).

(1) $$\mathrm{Cumulative}\;\mathrm{Release}\;(\%)=\frac{\mathrm{Acumulative}\;\mathrm{amount}\;\mathrm{of}\;\mathrm{LG}\;\mathrm{released}\;\mathrm{in}\;\mathrm{time}}{\mathrm{Initial}\;\mathrm{amount}\;\mathrm{of}\;\mathrm{LG}\;\mathrm{loaded}\;\mathrm{in}\;\mathrm{sample}}\times 100$$

Samples were analyzed in triplicates.

#### 2.3.2. Radical Scavenging Activity

Total antioxidant activity was measured with a stable free radical cation, (2,2′-azono-bis-3-ethylbenzthiazoline-6-sulphonate) diammonium salt (ABTS) [45]. A solution of 7 mM ABTS in distilled water was mixed with 2.45 mM potassium persulfate and held at room temperature for 12 h in the dark. The ABTS°^+^ radical cation solution was then diluted with phosphate buffer saline (PBS) medium (pH 7.4) and adjusted to obtain an absorbance of 0.70 ± 0.02 at 735 nm. Subsequently, 5 mL of the diluted solution was mixed with each weighed sample and left to stand for 30 min at room temperature in the dark. Absorbances of COL–CS–LG membranes, which reflected the quantity of ABTS radical cations remaining in the solution, were estimated using a spectrophotometer at 735 nm against the control. Following this, 160 µL ascorbic acid were dispersed into 5 mL ABTS solution and analyzed under the same conditions as the membranes. The radical scavenging activity (RSA) was calculated using Equation (2).

(2) $$\mathrm{RSA}\;\left(\%\right)=\frac{\left({\mathrm{Abs}}_{\mathrm{control}}-{\mathrm{Abs}}_{\mathrm{sample}}\right)}{{\mathrm{Abs}}_{\mathrm{control}}}\times 100$$

where RSA is the radical scavenging activity; Abs_control_ and Abs_sample_ correspond to the absorbance value of the control and the sample, respectively.

The results are expressed as mean values with their standard deviations indicated (mean ± SD).

#### 2.3.3. In Vitro Cytocompatibility Evaluation

In vitro cytocompatibility evaluation of the COL–CS–LG-based membranes was assessed using L929 murine fibroblasts purchased from the European Collection of Authenticated Cell Cultures (ECACC). All samples conditioned as films were cut into disks of 5 mm diameter and sterilized under UVC light for 4 h. Cells were grown in minimum essential medium (MEM; Sigma-Aldrich, Steinheim, Germany) supplemented with 10% fetal bovine serum (FBS; EuroClone, Milan, Italy) and 1% antibiotics (penicillin, streptomycin, and neomycin; Sigma-Aldrich, Steinheim, Germany) at 37 °C in a humidified atmosphere of 5% CO_2_. Cells were seeded at a density of 5 × 10^4^ cells/mL in 24-well tissue culture plates and incubated for 24 h to allow cell attachment. Then, the culture medium was replaced with fresh medium and samples were added into the wells (1 disk/well). Cells cultivated in fresh culture medium were used as controls. After 24 h and 72 h of cell incubation in the presence of the samples, quantitative ((3-(4,5-dimethylthiazol-2-yl)-2,5-diphenyltetrazolium bromide) (MTT) and lactate dehydrogenase (LDH) assays) and qualitative (cell morphology observations) analyses were performed.

The cell viability was evaluated via the 3-(4,5-dimethylthiazol-2-yl)-2,5-diphenyltetrazolium bromide (MTT) assay, as previously described by Stefan et al. [46]. Briefly, cells were incubated in 0.25 mg/mL MTT solution (Sigma-Aldrich, Steinheim, Germany) for 3 h at 37 °C. Then, the insoluble formazan crystals were dissolved with isopropanol, and the absorbance was measured at 570 nm using the SPECTROstar^®^ Nano microplate reader (BMG, Ortenberg, Germany). The amount of formazan was directly correlated to the number of metabolically active cells. The results are expressed as the percentage of viability compared to the negative control (untreated cells), which was considered 100% viable. Data are presented as the mean of three measurements ± standard deviation (SD).

The cytotoxicity of the samples was also evaluated by measuring the amount of lactate dehydrogenase (LDH) released into the culture medium. After 24 h and 72 h of treatment, 50 µL culture medium was used to perform the LDH assay using the CytoTox96 kit (Promega, Madison, WI, USA), according to the manufacturer’s instructions. The amount of LDH released into the culture medium was recorded at 490 nm using the SPECTROstar^®^ Nano microplate reader (BMG, Ortenberg, Germany). The obtained values were directly proportional to the number of lysed cells. Data are presented as a mean of three measurements ± SD.

Cell morphology was examined after 72 h incubation in the presence of the samples using Giemsa staining. Light micrographs of the L929 cells were obtained using a Zeiss Axio Observer D1 inverted microscope and analyzed with AxioVision 4.6 software (Carl Zeiss, Oberkochen, Germany).

#### 2.3.4. Gamma Irradiation and Chemiluminescence Spectroscopy

The γ irradiation process was performed by a γ irradiator M-38 GAMMATOR (USA), using a ^137^Cs source in air at room temperature at a dose rate of 0.4 kGy/h, COL–CS and COL–CS–LG 0.7% membranes being subjected to γ doses of 0, 10 and 25 kGy. Sterilization of heat-sensitive biomaterials for clinical applications is an easy and cost-effective treatment with advantages including less atmospheric pollution, due to the lack of chemical reagents, and high penetration power [47]. In a study performed by Susanto et al. [12], it was shown that via the gamma sterilization of chitosan–collagen membrane at a dose of 25 kGy, no microbial growth in the thioglycolate medium was observed until 14 days.

Gamma sterilization of collagen leads to the generation of hydroxyl radicals (°OH), superoxide anions (${O_{2}^{-}}^{{}^{\circ}}$) and radicals derived from amino acids. These radicals react to produce a free radical of collagen (${\mathrm{COL}}^{{}^{\circ}\;}$), a collagen crosslinked structure and peptides, according to Equations (3)–(5) [48]:

(3) $${O_{2}^{-}}^{{}^{\circ}}+\mathrm{COL}\to {\mathrm{COL}}^{{}^{\circ}}+{\;H}_{2}O_{2}/O_{2}$$

(4) $${\mathrm{COL}}^{{}^{\circ}\;}+{\;\mathrm{COL}}^{{}^{\circ}\;}\to \mathrm{COL}$$

(5) $${\mathrm{COL}}^{{}^{\circ}}+\mathrm{COL}\;\to \mathrm{peptides}\;+\mathrm{products}$$

In the case of chitosan, some studies have related that the gamma irradiation did not produce the crosslinking between the radicals produced by amino, alkyl, and hydroxyl groups, although some undetected crosslinking may exist [47,49].

The LUMIPOL 3 unit (SAS, Bratislava, Slovakia) chemiluminescence (CL) spectrometer was used for the recording of non-isothermal emission intensity dependence on increasing temperature for membrane samples of low weights, not exceeding 5 mg. For the CL measurements, small pieces of membrane containing 0.7% LG essential oil were placed in an aluminum cap, which was electrically heated in a micro-oven with programmed heating. The optimal heating rate (5 °C min^−1^) was applied. The selected temperature range started at room temperature and ends at 250 °C. The measured temperatures had low error (±0.5 °C). The CL intensity values were normalized to sample mass for reliable comparison.

#### 2.3.5. Attenuated Total Reflection–Fourier Transform Infrared Spectroscopy

The ATR–FTIR spectra were recorded on a Bruker VERTEX 70 spectrometer (Bruker, Ettlingen, Germany) with 4 cm^−1^ resolution. The background spectrum and sample spectra for non-irradiated COL–CS–LG membranes and irradiated COL–CS–LG 0.7% membranes were obtained in the 900–4000 cm^−1^ wavenumber range. Spectral processing was achieved with OPUS software (Bruker, Ettlingen, Germany).

#### 2.3.6. Differential Scanning Calorimetry

Thermal analysis of COL–CS and COL–CS–LG 0.7% membranes before and after gamma radiation was conducted using a DSC 823^e^ calorimeter supplied by Mettler Toledo (Greifensee, Switzerland), previously calibrated with indium standard. The samples, weighing between 8 and 10 mg, were packed in aluminum pans and placed in the DSC cell. The samples were heated from ambient temperature up to 220 °C at a rate of 10 °C min^−1^. The melting enthalpy (∆H_m_) and peak melting temperature (T_m_) were evaluated using the heating cycle.

#### 2.3.7. Minimum Inhibitory Concentration of Lemongrass Essential Oil

The minimum inhibitory concentration (MIC) of LG essential oil against *Staphylococcus aureus* ATCC 6538 and *Escherichia coli* ATCC10536 (Medclinic, Otopeni, Romania) was determined using the microdilution method, using gentamycin at 40 mg × mL^−1^ concentration as a reference. Microbial suspensions of 1.0 × 10^8^ CFU·mL^−1^ were added to 96-well plates with sterile broth (Muller Hinton agar) and binary dilutions of essential oil. The MIC values were determined by spectrophotometric measurements at 600 nm (Jasco 550, ABL&E-JASCO România S.R.L., Cluj Napoca, Romania) for each concentration, in triplicate, and repeated on at least three separate occasions.

#### 2.3.8. Antimicrobial Activity

The determination of antimicrobial properties of COL–CS and COL–CS–LG 0.7% membranes was performed in accordance with the provisions of the European Pharmacopoeia Edition 10/2020. The method consists of interacting the membranes with a concentration determined by the inoculum and determining their ability to reduce the initial concentration. For this purpose, bacteria or fungi were taken from the preserved stock to achieve the initial concentration. A plate with EA was striped, incubated at 37 °C ± 20 °C for 24 h to 48 h, and then 20 mL TSB was placed into a 100 mL Erlenmeyer flask. The initial cell concentration was previously determined by decimal dilutions (10^5^), and at the last dilution, for each strain, 100 µL were taken and spread onto nutrient agar. Plate counts were performed after 24 h incubation, which were then kept as a reference for cell growth in the control and test samples. Thus, plates with cell densities similar to that of dilution 10^5^ were considered to have similar CFU values (1.2 × 10^5^ CFU/mL for *Staphylococcus aureus*, 1 × 10^5^ CFU/mL for *Escherichia coli* and 2.5 × 10^4^ CFU/mL for *Candida albicans*). Subsequently, 1.0 ± 0.1 mL of the inoculum was pipetted onto several points over each test sample and then placed the sample in vials. After inoculation, the vials were shaken and 20 mL SCDLP medium was added immediately. The vials with the test material were placed in an incubator at (37 ± 1 °C) for 18–24 h, then 1 mL of the inoculum was taken from the bacterial suspension in the sample, placed in a test tube incorporating 9.0 mL ± 0.1 mL of NB and shaken well. Following this, 1 mL of this solution was added to a different test tube containing 9.0 mL ± 0.1 mL of medium and shaken well. The operations were successively repeated and a series of dilutions were prepared so that the dilutions were prepared 10 times in total. Subsequently, 1 mL of each dilution is pipetted into two Petri dishes, while 15 mL TSA was heated to 45 °C ± 1 °C in a water bath, added to the Petri dishes, and mixed well. The media were solidified at room temperature and incubated at 37 °C ± 2 °C for 24 to 48 h. Plate counts were performed at 24 h incubation in order to detect colony-forming cell units. To quantify the antimicrobial efficacy, the degree of microbial reduction (R, %) of each sample in triplicate was calculated, relative to the initial cell concentration. Briefly, after incubation, the number of colonies on the Petri dishes of the dilution series, on which 30 CFU to 300 CFU had appeared, were counted, and the bacteria concentration in the solution was obtained according to Equation (6):

(6) $$R=\frac{C_{t}-T_{t}}{C_{t}}\times 100\%$$

where $C_{t}$ is the average number of colonies of two control samples after 24 h, or the specified incubation period, expressed as CFU/mL; $T_{t}$ is the average number of colonies of two test samples after 24 h, or the specified incubation period, expressed as CFU/mL.

#### 2.3.9. Statistical Data

The biological results are expressed as the arithmetic mean ± standard deviation (SD) of the mean value, and one-way ANOVA conducted in Microsoft Excel was used to find the significance of the differences recorded between the membrane samples compared with the control. Values of the *p* coefficient (probability) < 0.05 indicate that there are statistically significant differences between the means of samples and the control.

## 3. Results and Discussion

### 3.1. Physicochemical Characterization of Hydrolyzed Collagen Powder and Collagen–Chitosan–Lemongrass Membranes

The processed collagen hydrolysate is a product without ash and with relatively high molecular weight, showing that the cleavage of the collagen was not very strong (Table 1). The lack of ash content proved that the potential of impurities was very low; accordingly, the collagen-based products for medical purpose requirements was fulfilled (ASTM F2212–19). The relatively high molecular weight of collagen hydrolysate processed by chemical and enzymatic hydrolyses allows one to preserve spinnable properties of native collagen in combination with chitosan polymers.

**Figure 1:**
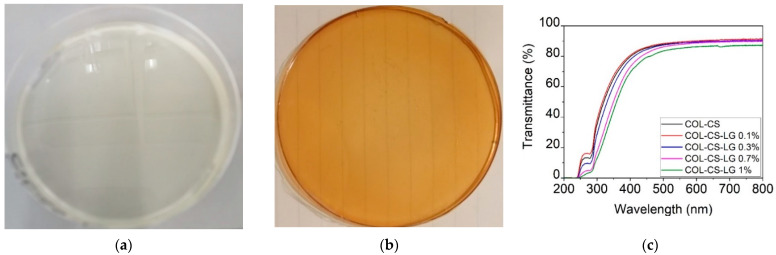
Visual appearance of COL–CS (**a**), COL–CS–LG 1% membranes (**b**), and transmittance in UV/VIS spectrometry of COL–CS–LG membranes (**c**).

The strong dispersion of LG essential oil 1% into COL–CS matrices can be observed in Figure 1a.

The transmittance of COL–CS–LG membranes (Figure 1b) increased with wavelength up to 400 nm, beyond which this optical property stabilized in the visible region. The addition of LG essential oil led to a slow decrease in the transmittance at 250−280 nm due to the hydroxyl auxochrome groups of chitosan [5]. The benefits associated with transparent wound dressing membranes can be associated with the easy monitoring of the wound and autolytic debridement, acting as an additional layer of the dressing as well as lowering risk of infections, thus, leading to the fast wound healing.

**Figure 2:**
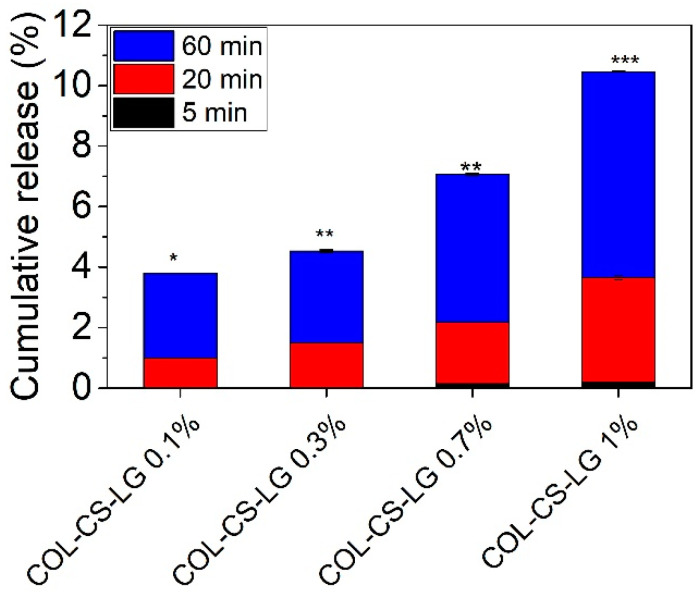
Cumulative release of LG essential oil from COL–CS–LG membranes. *, **, and *** mean *p* < 0.05, *p* < 0.01, and *p* < 0.001, respectively.

Figure 2 shows that the increase in LG essential oil loading from 0 to 1% led to enhanced cumulative release from COL–CS–LG membranes, indicating significant differences for the same group at *p* < 0.05, *p* < 0.01. Our study shows that the highest value of LG essential oil at 60 min is 10.46 ± 0.029% (*p* < 0.001), associated with the instant diffusion of LG essential oil from COL–CS matrices. This is advantageous to stop wound infections, control superficial scratches or wounds, and to manage wounds that are mildly exudative. This cumulative release of LG essential oil should provide the oxidative stability of COL–CS–LG membranes in medical applications. Another study reported a rapid cumulative release of 45% at t < 50 min for encapsulated LG essential oil into crosslinked chitosan via the emulsification–ionic gelation technique [50].

### 3.2. Antioxidant Activity Evaluation

The antioxidant ability of chitosan is attributed to the capacity of residual free amino groups to react with free radicals, generating macromolecular radicals with lower energy levels and ammonium groups [51]. As can be observed in Table 2, the antioxidant activity highly increased with the loading of LG essential oil. The mechanism of antioxidant activity can be explained, according to Mishra et al. [40], by free radical extraction of the hydrogen atom from LG essential oil and the transfer of an electron from the formed radical to the free radical. Additionally, antioxidant activity depends on the interactions between matrix components. It is supposed that the collagen–chitosan mixture has a synergic effect on the delivery of bioactive compounds, having the capacity to increase the antioxidant activity of COL–CS–LG membranes. The advantage of polymer membranes prepared by the casting method compared to nanospun collagen or nanofibers, where the radical scavenging activity decreases in time [40], is that the antioxidant activity increases with the LG essential oil concentration (Table 2).

### 3.3. In Vitro Cytocompatibility Evaluation of the Collagen–Chitosan–Lemongrass Membranes

In this study, the cytocompatibility of COL–CS–LG-based membranes was assessed by the MTT assay, which evaluates the activity of mitochondrial dehydrogenases, and by the LDH assay, which checks the cell membrane integrity by quantifying the levels of LDH enzyme released into the culture medium upon cell lysis.

**Figure 3:**
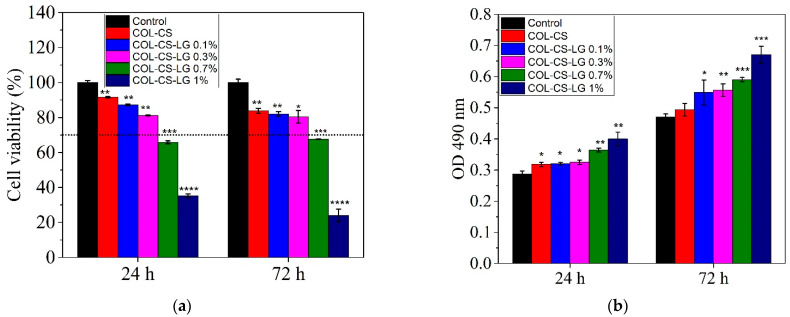
Viability of L929 murine fibroblasts cultivated in the presence of COL–CS–LG membranes for 24 h and 72 h, evaluated by MTT (**a**) and LDH (**b**) assays. Samples were compared to the control (cells cultivated in fresh culture medium), which was considered to have 100% viability. Data are expressed as mean values ± SD (*n* = 3). The horizontal dotted line at 70% to the graphic indicates the threshold. *, **, *** and **** mean *p* < 0.05, *p* < 0.01, *p* < 0.001, and *p* < 0.0001, respectively.

The MTT results show good cytocompatibility of the COL–CS and COL–CS–LG membranes containing 0.1% and 0.3% LG, whereas a higher quantity of LG (0.7% and 1%) induced a moderate or severe cytotoxic effect (Figure 3a). Throughout treatment, the membrane samples showed statistically significant differences compared to the control. Thus, after 24 h of treatment, the values of cell viability were higher than 70% (non-cytotoxic effect) for COL–CS (91.56%) (*p* < 0.01), COL–CS–LG 0.1% (87.24%) (*p* < 0.01) and COL–CS–LG 0.3% (81.28%) (*p* < 0.01), while for COL–CS–LG 0.7% this value slightly decreased (65.84%) (*p* < 0.001). Furthermore, COL–CS–LG 1% induced a significant decrease in cell viability (35.39%). Although cell viability slightly decreased after 72 h of treatment, the percentages were still maintained above 70% for COL–CS (83.93%) (*p* < 0.01), COL–CS–LG 0.1% (82.05%) (*p* < 0.01) and COL–CS–LG 0.3% (80.48%) (*p* < 0.05). Furthermore, the COL–CS–LG 0.7% exhibited very low cytotoxicity (67.80%) (*p* < 0.001), whereas COL–CS–LG 1% exhibited severe cytotoxicity (24.10%) (*p* < 0.001) (Figure 3a).

The integrity of the cell membrane was also evaluated by measuring the levels of LDH enzyme released into the culture medium. Our results show that the L929 murine fibroblasts cultivated in the presence of the tested films exhibited low levels of LDH released into the culture medium after 24 h and 72 h of treatment, similar to those of the control, for COL–CS, COL–CS–LG 0.1% (*p* < 0.05) and COL–CS–LG 0.3% (Figure 3b), suggesting no cytotoxic effect of these samples. However, the COL–CS–LG 0.7% and COL–CS–LG 1% membranes showed LDH levels up to 1.6 higher than those of the control, indicating a loss of the cell membrane integrity and, therefore, a cytotoxic effect.

**Figure 4:**
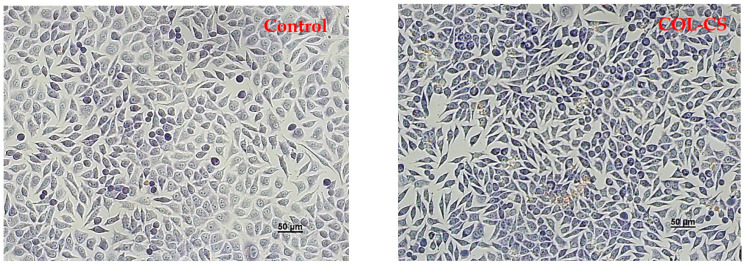

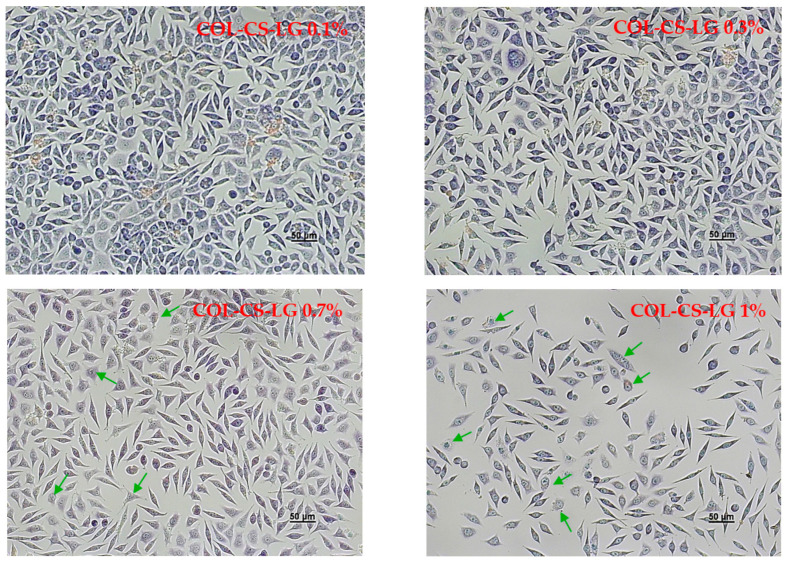
Light microscope images of L929 murine fibroblasts untreated (control) and treated with COL–CS; COL–CS–LG 0.1%; COL–CS–LG 0.3%; COL–CS–LG 0.7% and COL–CS–LG 1% for 72 h (Giemsa staining). Scale bar = 50 µm. Green arrows indicate some examples of abnormal cells.

Light micrographs of L929 murine cells cultivated in the presence of the films revealed normal cell morphology, similar to that of the control, for COL–CS, COL–CS–LG 0.1% and COL–CS–LG 0.3% (Figure 4). Cells exhibited a fibroblast-like phenotype, with clear cytoplasm, cytoplasmic extensions and euchromatic nuclei with multiple nucleoli. Cell density was also similar to that of the control, reaching an almost complete monolayer, with cells covering around 85–90% of the well surface. Some morphological changes in cell shape (rounded and stellate cells) and cytoplasm appearance were observed for COL–CS–LG 0.7%, whereas COL–CS–LG 1% membranes induced more changes in cell morphology together with a significant decrease in cell density (Figure 4).

In the present study, the in vitro cytocompatibility of the COL–CS–LG membranes was evaluated by two quantitative assays, MTT and LDH tests, and by one qualitative analysis, cell morphology observations from Giemsa staining. Our results indicate a good degree of cytocompatibility of the COL–CS membrane and those containing 0.1% and 0.3% LG, with cell viability values higher than 80% after 72 h of treatment. However, the increase in the amount of LG has led to a moderate and even severe cytotoxicity, in the case of COL–CS–LG 0.7% and COL–CS–LG 1% membranes, respectively. Furthermore, the cell morphological observations are in line with the quantitative results obtained using biochemical assays. In the literature, very few studies have reported results from in vitro biocompatibility tests on biomaterials containing LG essential oil. For instance, Liakos et al. [36] created composite fibrous scaffolds based on cellulose acetate and 5% (*v*/*v*) LG essential oil using an electrospinning technique for use as wound dressings, and proved their non-cytotoxicity on two cell lines directly involved in the wound healing process (fibroblasts and human keratinocytes). High cell viability and biocompatibility potential was also reported for functional nanocapsules made of polylactic acid (PLA) and LG essential oil (5% *v*/*w*) in order to be used for the design of efficient, personalized and ecological drug delivery systems with considerable impact in antimicrobial therapy [52]. In addition, chitosan–alginate nanocarriers developed for the encapsulation of LG essential oil (0.2 and 0.4 mg/mL) proved to have no cytotoxic effect when tested on A549 human lung adenocarcinoma epithelial cell line [41]. Thus, between concentrations of LG essential oil ranging from 0.008 to 0.03% (*v/*v**), cell viability was above 80% and the cytotoxic effect was reported at 0.125% (*v/*v**) [31]. However, further studies are needed regarding the use of different methods for cell viability assays.

### 3.4. Non-Isothermal Chemiluminescence

**Figure 5:**
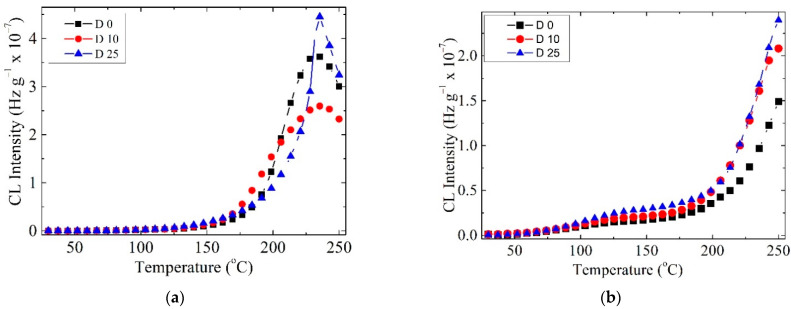
CL spectra recorded at doses up to 25 kGy for the COL–CS membrane (**a**) and COL–CS–LG 0.7% membrane (**b**).

The thermal degradation of the investigated samples showed variable non-isothermal CL spectra, due to the presence of unstable organic structures from lemongrass. This component, which contains citral (3,7-dimethyl-2,6-octadienal), slows down oxidation at temperatures exceeding 200 °C. The COL–CS sample presents a deceleration of oxidation, when the specimens are subjected to the action of γ irradiation (Figure 5a). This requires the protection of polymeric membranes during the radio-sterilization process by the beneficial action of LG essential oil. Whilst oxidation in the LG-free samples was initiated at medium temperature (100 °C), earlier degradation of the patterns with LG starts at 50 °C, and the progress of oxidation is slowly achieved (Figure 5b). However, the presence of this ketonic structure, an electronegative moiety attracting free radicals possessing unpaired electrons, as well as the possible involvement of structural unsaturation of citral hindering the growing of CL emission, illustrate its delaying effect on the propagation of oxidation. When compared with COL–CS membranes subjected to irradiation, it is observed that the 0.7% LG essential oil contribution leads to decreased CL intensity.

Radio-sterilization of pharmaceutical products or biological tissues by gamma irradiation with a dose up to 25 kGy is recommended by the ISO 11137 standard, without any biological validation [47]. Studies have reported the stabilization by γ irradiation of polymeric materials due to the natural antioxidants, such as vanillic acid, caffeic acid [53] and rosemary ethanolic extract [54].

Data on the delaying effect of LG essential oil on the generation of radicals by the COL–CS–LG 0.7% membrane during irradiation at different doses are presented in the Supplementary Materials, Figure S1.

### 3.5. Infrared Spectroscopy Analysis

**Figure 6:**
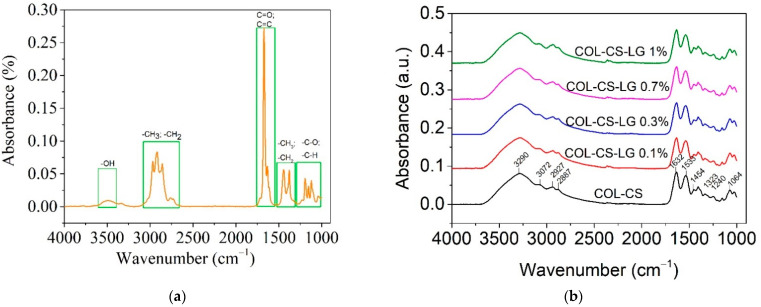
ATR–FTIR for LG essential oil (**a**) and COL–CS–LG membranes with different content of LG essential oil (**b**).

Figure 6 shows the ATR–FTIR spectra of LG essential oil and the COL–CS–LG membranes.

The LG essential oil spectra show the characteristic absorption bands in the range of 2731 cm^−1^ to 2970 cm^−1^, denoting the -CH=O stretching, asymmetric stretching of -CH_3_ and symmetric and asymmetric stretching of -CH_2_ (Figure 6a). The intense peak at 1672 cm^−1^ is associated with C=O bonds arising from the two aldehydes of neral and geranial [40,50,52]. The low-intensity band observed at 1722 cm^−1^ is due to vibrations of C=C (cis and trans), confirming the presence of conjugated double bonds (C=C-CHO) in citral, which are common in acyclic monoterpenes [41], while the peak at 1629 cm^−1^ indicates the stretching of C=C of the aldehyde group. The bands at 1438 cm^−1^ and 1375 cm^−1^ were ascribed to -CH_3_ vibration absorption and the bending of the -CH_2_ group. From 1195 to 1045 cm^−1^, the stretching of -C-O and the vibrations of the CH skeleton were observed. Similar peaks were previously reported for other species of *Cymbopogon* [55].

The COL–CS membrane exhibited absorption peaks localized at 3290 cm^−1^ and 3072 cm^−1^ (amide B); 2927 cm^−1^ and 2887 cm^−1^ (symmetric or asymmetric stretching vibration of CH_2_ group), 1632 cm^−1^ and 1533 cm^−1^ (C=O vibration of amide I band), 1454 cm^−1^ (C-H bending vibration), 1400 cm^−1^ (OH groups bending), 1323 cm^−1^ (-CH_3_ in amide group), 1240 cm^−1^, 1147 cm^−1^ (-C-O-C- in glycosidic linkage of chitosan), 1197 cm^−1^, 1064 cm^−1^ and 1029 cm^−1^ (stretching vibrations of C-O-C in chitosan) (Figure 6b). These bands are assigned to chitosan [10] and collagen [56], where the band at 1238 cm^−1^ is characteristic of amide III [19]. The overlapping stretching vibration absorption peaks ranging from 2887–3290 cm^−1^ appears to be due to the vibration of the -NH_2_ and -CH stretching of individual chitosan and collagen biopolymers [12,57].

The introduction of LG essential oil into COL–CS matrices did not change the positions of characteristic peaks (Figure 6b). The intensity of absorption peaks at 2927 cm^−1^ can be assigned to the LG essential oil, while the specific peaks of LG essential oil at 1629 cm^−1^ overlap with those of the COL–CS structure. Other studies have also highlighted the presence of bioactive compounds from LG essential oil into cellulose acetate wound dressings [36] and chitosan nanoparticles [50].

**Figure 7:**
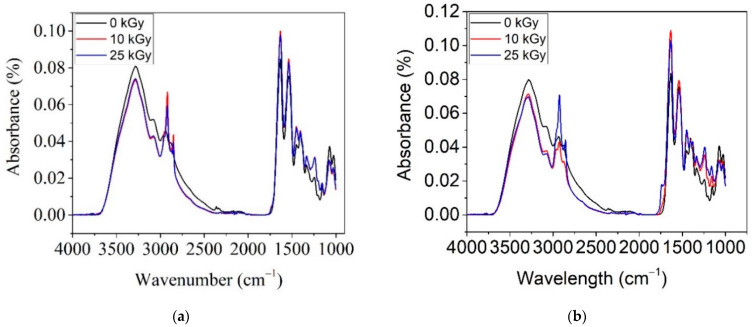
ATR–FTIR spectra for irradiated COL–CS (**a**) and COL–CS–LG 0.7% (**b**).

Figure 7 shows the ATR–FTIR spectra for COL–CS and COL–CS–LG 0.7% irradiated with 0, 10 and 25 kGy. The purpose of this analysis is to determine whether the irradiation process induces significant changes in the membrane structure.

From Figure 7a, for the COL–CS membrane, one can observe the decrease in intensity of band at 3290 cm^−1^ with irradiation dose, signifying the degradation of the amide group. The same effect of decreasing the peak intensity with the applied dose was also reported by [58]. A contrary effect of increased band intensity, was observed for peaks at 2923 cm^−1^, 2887 cm^−1^, 1632 cm^−1^, 1533 cm^−1^, 1454 cm^−1^, 1400 cm^−1^, 1323 cm^−1^ and 1240 cm^−1^. This behavior is due to the beneficial effect of irradiation on the crosslinking of the macromolecular chain of biopolymers. It is known that at a low gamma irradiation dose, the crosslinking effect predominates, whilst at a high dose, scission occurs in acid group polymers. The competition between crosslinking and scission depends on polymer nature, dose rate as well as dosage.

The spectra of irradiated COL–CS–LG 0.7% membranes up to 25 kGy reveal a slight decrease in absorption bands at 3273 cm^−1^, 3076 cm^−1^, 1066 cm^−1^ and 1020 cm^−1^, together with the shifting of bands at 3276 cm^−1^ to 3288 cm^−1^ (Figure 7b). An increase in the absorption peaks due to irradiation is observed at 2927 cm^−1^, 2854 cm^−1^ and 1465 cm^−1^ for the applied dose of 25 kGy, indicating the formation of CH groups at 1637 cm^−1^, 1328 cm^−1^ and 1240 cm^−1^ for all doses, and at 1533 cm^−1^ for the membrane irradiated with 10 kGy. The band at 1145 cm^−1^ for non-irradiated membranes shifted to 1164 cm^−1^ and increased in intensity in the irradiated membranes, indicating bond strengthening and as well as conformational changes. The FTIR spectra of the membranes without LG are almost identical at both gamma irradiation doses whilst the gamma irradiated LG-containing membranes have differentiated FTIR spectra indicating the influence of LG on the radiation induced modifications. The most obvious difference is the intensity of the antisymmetric CH stretch peak, which increases for 10 kGy LG-free membranes, respectively, for 25 kGy LG-containing membranes. The increase in intensity of CH bands is correlated with scission phenomena, thus confirming the membrane radiation protection of LG essential oil.

### 3.6. Thermal Analysis

**Figure 8:**
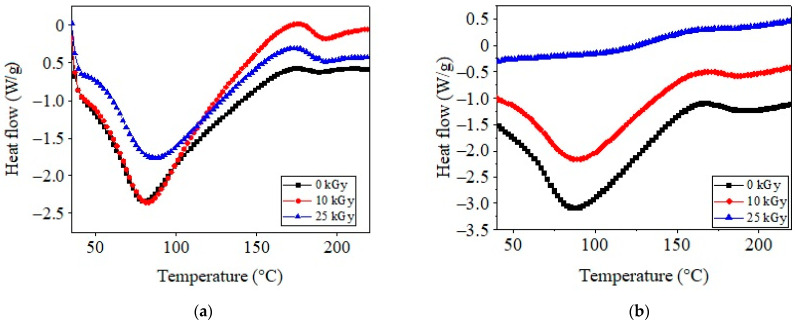
DSC curves for COL–CS (**a**) and COL–CS–LG 0.7% membranes (**b**).

DSC spectra for COL–CS and COL–CS–LG 0.7% membranes subjected to irradiation are shown in Figure 8a,b.

As shown in Figure 8a, the COL–CS membrane produced an initial endothermic peak at around 80.7 °C, which appeared due to the release of free and bonded water from the matrices [5,56,59]. The irradiation of COL–CS and COL–CS–LG 0.7% membranes led to shifts in the water evaporation threshold to higher temperatures (83.4, 88.1 and 88.7 °C, respectively). The second thermal event is associated with the thermal degradation or denaturation of CS and COL, occurring at 188.1 °C for the COL–CS membrane and 190.7 °C for the COL–CS–LG 0.7% membrane. It should be noted that the thermal stability of the COL–CS–LG 0.7% membrane improved by 1.4 °C. Additionally, from Figure 8b it can be observed that the irradiation method had a crosslinking effect on the COL–CS membrane, clearly marked by the increase in the decomposition temperature to around 200 °C as compared with non-irradiated samples (190.7 °C). Other studies have reported the thermal parameters for chitosan at 68.5 °C and 175.1 °C, while for collagen they have been recorded at 63.9 °C and 226.8 °C [59].

### 3.7. Minimum Inhibitory Concentration Determination

The values of minimum inhibitory concentrations against Gram-positive and Gram-negative bacteria (0.156 *v*/*v*% and 0.039 *v*/*v*%) fall within the non-cytotoxic concentrations for cell viability (0.1 *v*/*v*% and 0.3 *v*/*v*%), as Table 3 and Figure 3 show, which ensures antimicrobial efficiency without harmful effects.

Similar values for minimum inhibitory concentrations for LG essential oil in the range of 0.25% (*v*/*v*) and 1% (*v*/*v*) were reported against Gram-negative drug-resistant *A. baumannii* [20]. Other previous studies showed that the LG essential oil in concentrations of 0.03% (*v*/*v*) and 0.06% (*v*/*v*) was effective at inhibiting the growth of *S. aureus* [60], while more recent studies found that concentrations of 0.07% (*v*/*v*) are efficient and 0.31% (*v*/*v*) is needed for combinations of *S. aureus* and *C. albicans*. MIC values of 0.19, 0.39 and 3.12 mg × mL^−1^, were also reported for the antibacterial strength of pure LG essential oil against *C. albicans, S. aureus* and *E. coli*, respectively [50]. The mechanism of antimicrobial activity of LG essential oil was attributed to the citral component, with efficiency in reducing *agrA* and α-toxin-encoding gene (hla) expression, responsible for lethality [33]. Other effects of citral have been related to the suppression of genes responsible for fatty acid and peptidoglycan biosynthesis in *S. aureus* [33].

### 3.8. Antimicrobial Activity

The results of the antimicrobial tests against Gram-negative, Gram-positive and a fungus strains are presented in Table 4 and showed efficiency above 99% in all cases. The results are in agreement with other studies related to *S. aureus* and *Candida albicans* antibiofilm properties of lemongrass essential oil at 0.3125% concentration [33]. This previous study underlines the importance of dual resistant antimicrobials due to the coexistence of fungal and bacterial strains with improved resistance to conventional antibiotics. The biofilm of *S. aureus* grown with *Candida albicans* is more developed and resistant to vancomycin and oxacillin antibiotics, contributing to diseases with high morbidity [33].

The introduction of LG essential oil into COL–CS membranes slightly decreased the antibacterial effect (Table 4). This behavior can be explained by the difficulty of microorganism tests that are able to penetrate the COL–CS–LG 0.7% membrane to reach the essential oil’s active biocompounds, due to the dimension of microorganisms as well as the low release of LG essential oil from matrices. Other authors suggest that a synergism between citral and geraniol decreases the antimicrobial activity [39], while no antimicrobial properties against *Escherichia coli* and Staphylococcus aureus were reported in the case of chitosan and collagen–chitosan–hyaluronic acid films [59].

## 4. Conclusions

The influence of LG essential oil on the structure of COL–CS–LG membranes was highlighted by FTIR spectroscopy and DSC analyses. The high antioxidant and essential oil cumulative releasing properties of COL–CS–LG membranes show the potential for using 1% essential oil, while the non-cytotoxic concentration of 0.3% (*v*/*v*) and very low cytotoxic concentration of 0.7% (*v*/*v*) are in agreement with MIC values for *Escherichia coli* and *Staphylococcus aureus*.

The original results of chemiluminescence spectroscopy analyses of COL–CS–LG 0.7% membranes after exposure to 0, 10 and 25 kGy show, for the first time, the protective role of the citral component of lemongrass essential oil at temperatures above 200 °C, associated with the antioxidant property and slight crosslinking of COL–CS components revealed through DSC analysis.

Antimicrobial efficiency against Gram-positive and Gram-negative bacteria, and an opportunistic pathogenic yeast, was found to be above 99.60%, and allows us to recommend the COL–CS–LG 0.7% membrane for potential use in biomedical applications.

## Figures and Tables

**Table 1:** Collagen hydrolysate characteristics.

Characteristics	U.M.	Values ± SD
Volatile matter	%	10.67 ± 0.35
Total ash	%	Non-detectable
Total nitrogen	%	16.74 ± 0.35
Protein	%	94.06 ± 0.35
Aminic nitrogen	%	0.65 ± 0.14
Molecular weight	Da	22,500 ± 500
Conductivity (10% solution)	μS/cm	0.57 ± 0.10
pH (10% solution)	pH units	4.40 ± 0.10

**Table 2:** ABTS radical scavenging activity of COL–CS and COL–CS–LG membranes and the positive control (*p* < 0.05 between groups).

Sample	RSA (%)
COL–CS	16.25 ± 0.14
COL–CS–LG 0.1%	27.50 ± 0.10
COL–CS–LG 0.3%	77.84 ± 0.21
COL–CS–LG 0.7%	79.69 ± 0.23
COL–CS–LG 1%	84.07 ± 0.18
Ascorbic acid	87.24 ± 0.12

**Table 3:** MIC against *E. coli* and *S. aureus* of LG essential oil.

Bacteria Strain	MIC, mg × mL^−1^	
	LG Essential Oil	Gentamicin
*E. coli*	1.56 ± 0.02	0.07 ± 0.02
*S. aureus*	0.39 ± 0.01	0.05 ± 0.01

**Table 4:** Antimicrobial activity of COL–CS-LG 0.7% membrane at 48 h.

Film	R (%)		
	*E. coli*	*S. aureus*	*C. albicans*
COL–CS	100	100	100
COL–CS–LG 0.7%	99.81 ± 0.021	99.92 ± 0.015	99.65 ± 0.032

## Data Availability

The data presented in this study are available on request from the corresponding author.

## Supplementary Materials

The following supporting information can be downloaded at: https://www.mdpi.com/article/10.3390/polym14183884/s1. Figure S1. Influence of lemongrass (0.7%) on the generation of radicals for COL–CS film with temperature at doses of 0 kGy (**a**), 10 kGy (**b**) and 25 kGy (**c**).
