# Supplementary material for: Bioactivity and Thermal Stability of Collagen–Chitosan Containing Lemongrass Essential Oil for Potential Medical Applications
Source: Polymers (Basel). 2022 Sep 17;14(18):3884. doi: 10.3390/polym14183884 (PMC9503703; doi:10.3390/polym14183884)
Supplement: Supplementary file 1 [file polymers-14-03884-s001.zip › polymers-1919274-supplementary.pdf]

polymers

Supplementary Materials

Bioactivity and Thermal Stability of Collagen–Chitosan Containing Lemongrass Essential Oil for Potential Medical Applications

Maria Râpă [1], Traian Zaharescu [2], Laura Mihaela Stefan [3], Carmen Gaidău [4,*], Ioana Stănculescu [5,6,*], Rodica Roxana Constantinescu [4] and Maria Stanca [4]

1 Faculty of Materials Science and Engineering, University Politehnica of Bucharest, 313 Splaiul Independentei, 060042 Bucharest, Romania
2 INCDIE ICPE CA, 313 Splaiul Unirii, P.O. Box 149, 030138 Bucharest, Romania
3 National Institute of R&D for Biological Sciences, 296 Splaiul Independentei, 060031 Bucharest, Romania
4 The National Research & Development Institute for Textiles and Leather-Division Leather and Footwear Resesarch Institute (ICPI), 93 Ion Minulescu Street, 031215 Bucharest, Romania
5 Horia Hulubei National Institute of Research and Development for Physics and Nuclear Engineering, 30 Reactorului Street, 077125 Magurele, Romania
6 Department of Physical Chemistry, University of Bucharest, 4–12 Regina Elisabeta Boulevard, 030018 Bucharest, Romania
* Correspondence: carmen_gaidau@hotmail.com (C.G.); istanculescu@nipne.ro (I.S.)

Citation: Râpă, M.; Zaharescu, T.; Stefan, L.M.; Gaidău, C.; Stănculescu, I.; Constantinescu, R.R.; Stanca, M. Bioactivity and Thermal Stability of Collagen–Chitosan Containing Lemongrass Essential Oil for Potential Medical Applications. *Polymers* **2022**, *14*, 3884. https://doi.org/10.3390/polym14183884

Academic Editor: Swarup Roy

Received: 31 August 2022
Accepted: 13 September 2022
Published: 17 September 2022

Publisher's Note: MDPI stays neutral with regard to jurisdictional claims in published maps and institutional affiliations.

Copyright: © 2022 by the authors. Submitted for possible open access publication under the terms and conditions of the Creative Commons Attribution (CC BY) license (https://creativecommons.org/licenses/by/4.0/).

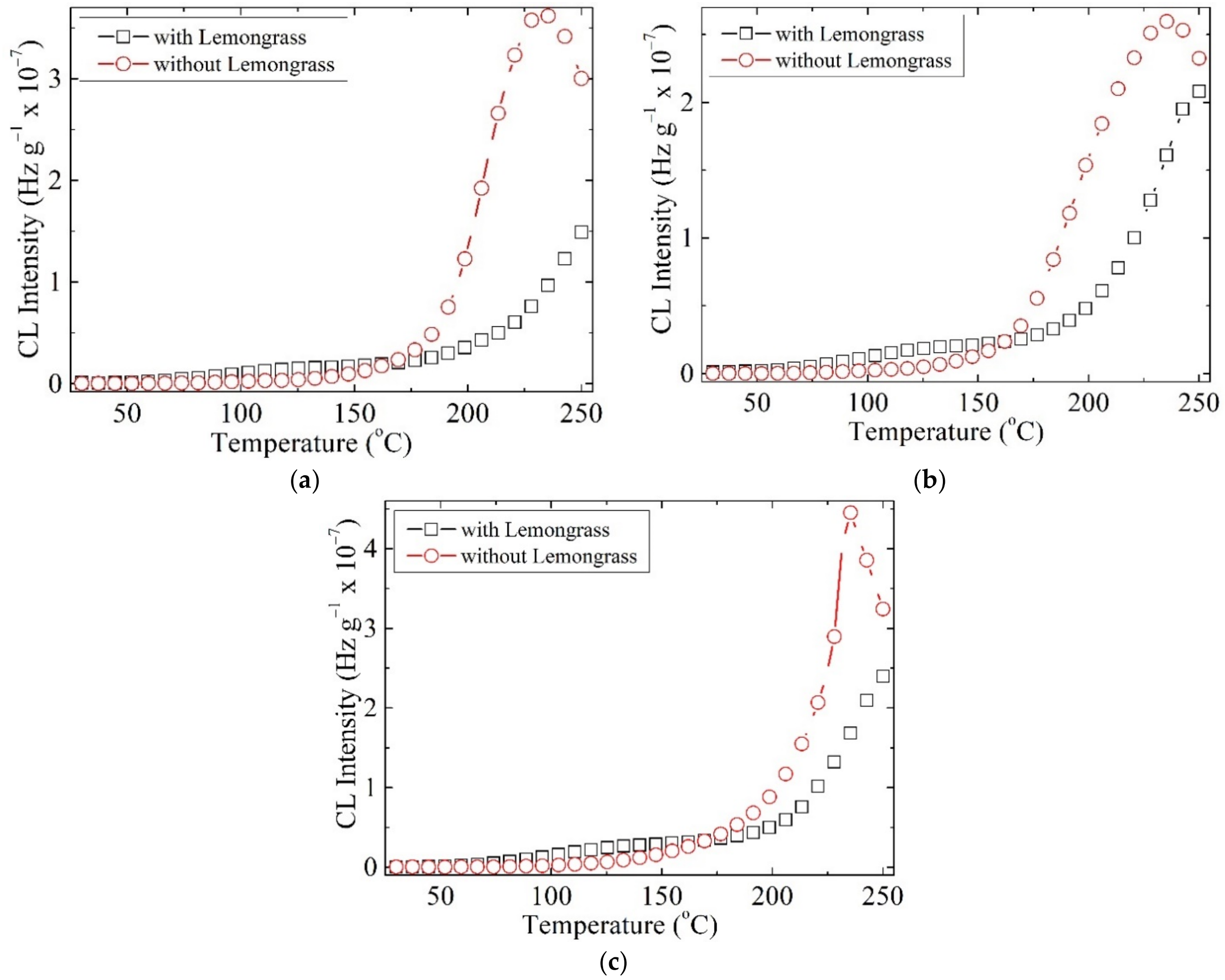

Figure S1. Influence of lemongrass (0.7%) on the generation of radicals for COL/CS film with temperature and dose. 0 kGy (**a**); 10 kGy (**b**); 25 kGy (**c**).
